# Recurrent Amyloid Material in Grafts Used in Patients with Lattice Corneal Dystrophy 2 (Meretoja’s Syndrome)

**Published:** 2014

**Authors:** Valentín Huerva, Jordi Soldevila, Xavier Matias-Guiu

**Affiliations:** 1 Ophthalmology Department, Universitary Hospital Arnau de Vilanova, Lleida, University of Lleida, IRB LLEIDA, Spain; 2 Pathology Department, Universitary Hospital Arnau de Vilanova, Lleida, University of Lleida, IRB LLEIDA, Spain

**Keywords:** LCD Type 2, Recurrent Amyloid Material, Grafts, Meretoja’s Syndrome

**Dear Editor**

**Figure 1:**
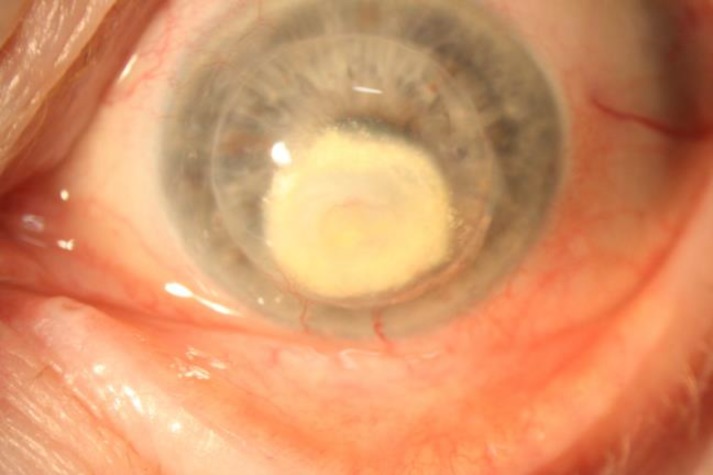
White deposits in the corneal button 8 years after keratoplasty in LCD2.

The lattice corneal dystrophies (LCD) are characterized by an accumulation of amyloid within the cornea. There are several types of LCD, and in the LCD2 type the corneal features are subtly unique. The lattice lines are finely arranged and tend to be denser in the periphery (1,2). Corneal erosions occur less frequently, and visual acuity is preserved until the sixth decade (2). Lattice corneal dystrophie 2 is associated with systemic amyloidosis type V (Meretoja syndrome/Finnish type) (1-4). This is a systemic autosomal dominant disease that arises in early adulthood and predominantly affects the cornea, skin, and cranial nerves (2,3). Amyloid deposition corresponds to a degradation product of gelsolin. Amyloidosis type V was first described in Finland (3) where it occurs with high frequency (1). New cases have been reported in several other countries (5). Regular biomicroscopic examination and intraocular pressure measurements are recommended since the disease is incurable and amyloid deposition will continue. When significant central corneal haze is present, only keratoplasty can clarify the cornea and restore vision. However, the graft could accumulates new amyloid. Slight-documentation of these grafts has been reported to survive. We recently reoperated on a Spanish patient with Meretoja syndrome (5). The patient suffered a graft rejection two years after a corneal transplant in the left eye. Months later a neurotrophic central defect was present in the cornea with progressive accumulation of a white substance suspected to be amyloid material (Fig. 1).

**Figure 2:**
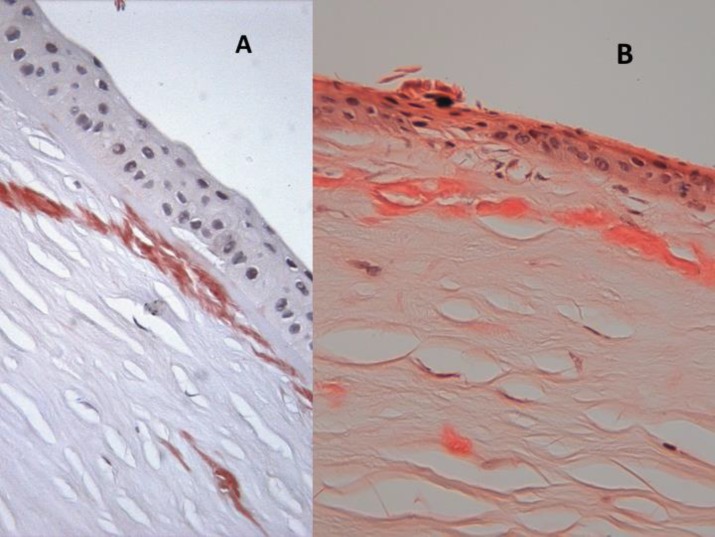
Congo red shows deposition of material under Bowman´s layer in LCD2 (A).

Recurrent amyloid in the corneal button in the same pattern as previously (B).

Biomicroscopical examination showed a white area surrounding the corneal vessels similar in appearance to the lipid leakage from corneal stromal vessels (Fig.1). Vascular leakage could have led to amyloid deposition, rather than real dystrophy recurrence. 

Given the total failure of the graft, a new penetrating keratoplasty was decided. The procedure was performed without complications in March 2014, 8 years after the first keratoplasty. 

Histology of the corneal button showed fibrosis and changes secondary to the previous surgical procedure. Subepithelial deposits of amyloid were seen with a pattern identical to that of the first surgical specimen (Fig. 2). In LCD2, the amyloid marker Congo red will show deposition of material under Bowman’s layer and in some cases at the level of the epithelial basal membrane. The disease is under-reported. Originally, the material was believed to arise from degenerated nerves but now it is believed to be locally produced amyloid (6). Some authors feel that the disease may recur in the graft (1). According to our observations, the amyloid tends to recur in the grafted button with a similar distribution. Despite graft rejection, progressive amyloid accumulation under Bowman’s membrane may contribute to graft failure. Our findings suggest that in LCD2, keratoplasty is not recommended until vision is indigent because the disease may recur after a short time and graft survival will be limited to a few years.
